# Lumbar decompression and fusion for symptomatic spinal stenosis in a patient with chronic thoracic sensory level from prior transverse myelitis: a case report

**DOI:** 10.1186/s13256-024-04367-9

**Published:** 2024-02-04

**Authors:** Benjamin D. Pesante, Mitch R. Paro, Tooba Nadeem, Ketan R. Bulsara, David B. Choi

**Affiliations:** 1 University of Connecticut School of Medicine, UConn Health Center, 263 Farmington Ave, Farmington, CT 06030 USA; 2 University of Connecticut, Storrs, CT USA; 3 Division of Neurosurgery, Department of Surgery, UConn Health, Farmington, CT USA

**Keywords:** Case report, Transverse myelitis, Spinal stenosis, Lumbar decompression

## Abstract

**Background:**

Many patients with transverse myelitis suffer from sensory loss below the spinal level of the lesion. This is commonly associated with chronic neuropathic pain. However, the presence of somatic pain below a complete thoracic sensory level after transverse myelitis is exceptionally rare, and it is unclear if surgical decompression is an effective form of treatment for these patients.

**Case presentation:**

In this report, we describe a 22-year-old Caucasian female who suffered from chronic lumbar back pain despite a complete thoracic sensory level secondary to prior transverse myelitis. Imaging demonstrated multilevel central stenosis below the sensory level, and her pain improved after surgical decompression. To our knowledge, this is the first reported case of symptomatic lumbar stenosis below a sensory level after transverse myelitis successfully treated with surgical decompression.

**Conclusion:**

This is the first reported case of a patient with symptomatic lumbar stenosis after transverse myelitis whose lower back pain and quality of life improved following surgical decompression and fusion. This case provides evidence that typical lumbago is possible in patients with sensory loss from transverse myelitis, and standard lumbar decompression may provide benefit for these patients.

## Background

Transverse myelitis (TM) is an acute inflammatory disorder of the spinal cord, presenting with rapid-onset autonomic, sensory, and motor impairment below the level of inflammation [1]. The cause of TM is unknown in most cases, but is often associated with infection or autoimmune disease [1, 2]. Symptoms include sensory alteration, weakness, autonomic dysfunction, and temperature dysregulation [3]. In the acute phase, 50% of patients are paraplegic, and almost all have bladder or bowel dysfunction. Approximately 33% of patients recover with little to no lasting deficits, 33% have a moderate degree of permanent disability, and 33% are permanently disabled [4]. Recovery is unlikely if symptoms do not improve within the first 3–6 months.

Lumbar spinal stenosis is a pathologic narrowing of the intraspinal canal, lateral recess, or neural foramina, most often secondary to spondylosis. The condition typically affects individuals over age 60 [5]. Symptoms include low back pain, neurogenic claudication, sensory disturbance, paresis, and bowel/bladder dysfunction. Surgical treatment is usually performed if conservative treatment fails. Surgical approaches include single or multilevel decompressive laminectomy with or without lumbar fusion. Roughly 23% of patients that undergo surgery for lumbar spinal stenosis need a subsequent surgical procedure [6].

Here, we describe a patient with a remote history of thoracic transverse myelitis with resultant paraplegia and sensory loss presenting with chronic low back pain that improved after lumbar laminectomies for lumbar spinal stenosis.

## Case presentation

**Fig. 1:**
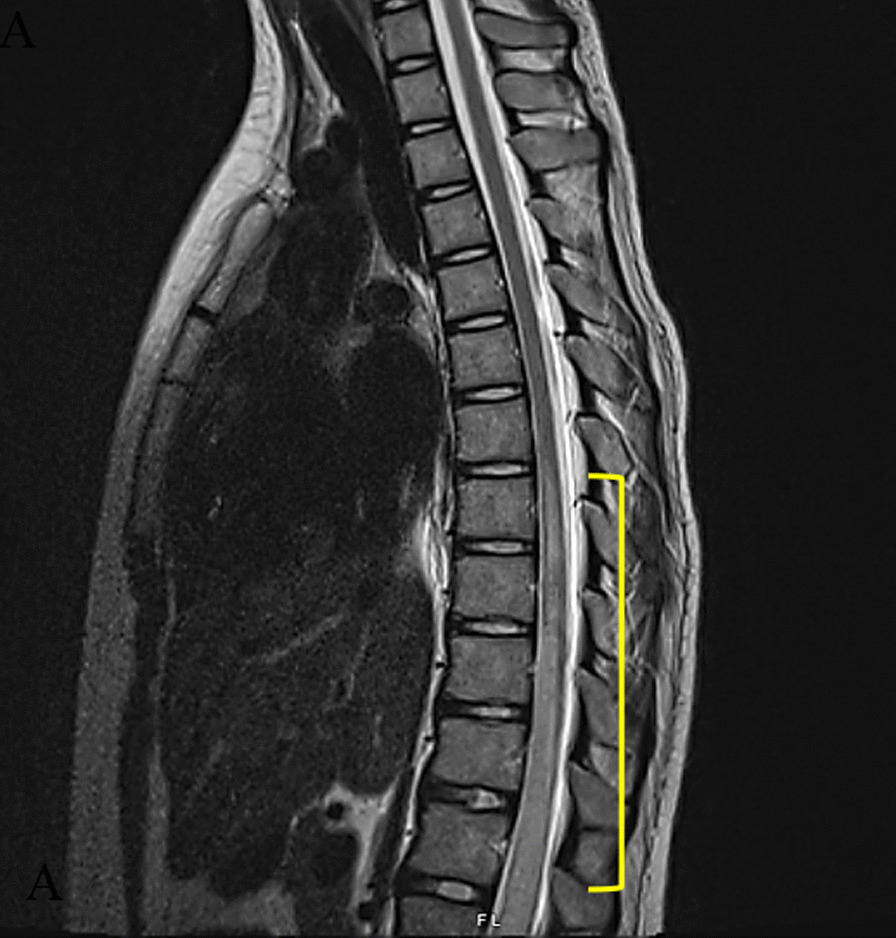
Image obtained at time of diagnosis of TM. T2-weighted sagittal MRI of thoracic spine demonstrating signal abnormality without abnormal enhancement extending from T7 to L1 with cord edema and expansion most prominent at T9–L1 (bracket)

A 22-year-old Caucasian female with a remote history of transverse myelitis presented with 3 years of worsening midline low back pain. The episode of transverse myelitis occurred 8 years prior to presentation (Fig. 1). At that time, she suffered paraplegia, bowel and bladder incontinence, complete sensory loss localized to the T8 level, diffuse pains and paresthesia in the upper extremities, and low back pain below the sensory level. One and a half years after the initial episode of TM, the patient reported a pain burden score (PBS) in her low back below the sensory level of 22/28. She also reported loss of function and inability to do transfers, indicating worsening quality of life.

Due to worsening lower back pain, loss of function, and development of neuromuscular scoliosis secondary to TM, the patient decided to undergo posterior spinal fusion T3 through pelvis. Preoperatively, in addition to low back PBS score of 22/28, her lower extremity physical exam was notable for complete paraplegia with wheelchair bound status and inability to ambulate, atrophy and edema in all muscles in the lower extremities, a sensory level of T10 on the right and T8 on the left, with only some patchy sensation to pressure evident over her left thigh but otherwise no neuropathic pain or other sensation in the lower extremities bilaterally, and absence of lower extremity reflexes. She underwent surgery of posterior spinal fusion T3 through pelvis which was complicated by wound infection and subsequent incision and debridement. She was discharged following a 60-day hospital stay, and 6 months postoperative the patient reported improved low back pain with PBS 13/28 and unchanged motor and sensory exams in the lower extremities. At this time, she also reported improved mood and ability to conduct activities of daily living, indicating improved quality of life compared with prior to surgery. Unfortunately, at 1.5 years post spinal fusion T3-pelvis, the patient presented to clinic with worsening lumbar back pain and PBS increased to 22/28, worsened with prolonged sitting. Bilateral loosening of the pelvic fixation screws were demonstrated on imaging and deemed to be the cause of the patient’s pain as a result of increased load at the L4–S1 levels. She underwent revision of the pelvic fixation, which improved her PBS to 18/28. The postoperative course was complicated by a new MRSA infection at the incision site and recurrent wound dehiscence, leading to seven incision and drainage treatments over a 6-month duration. The patient then underwent removal of segmental instrumentation T3-pelvis, which initially improved back pain to PBS score 12/28. The patient’s quality of life had clearly increased, as she reported doing remarkably well with increased mobility. Additionally, her mother reported the patient was more functionally active than she had been in a year. Unfortunately, her low back pain continued to progressively worsen over the next 3 years, with lower back pain increasing to PBS score 15/28, at which point she approached our team to consider redo L4–S1 decompression and fusion.

**Fig. 2:**
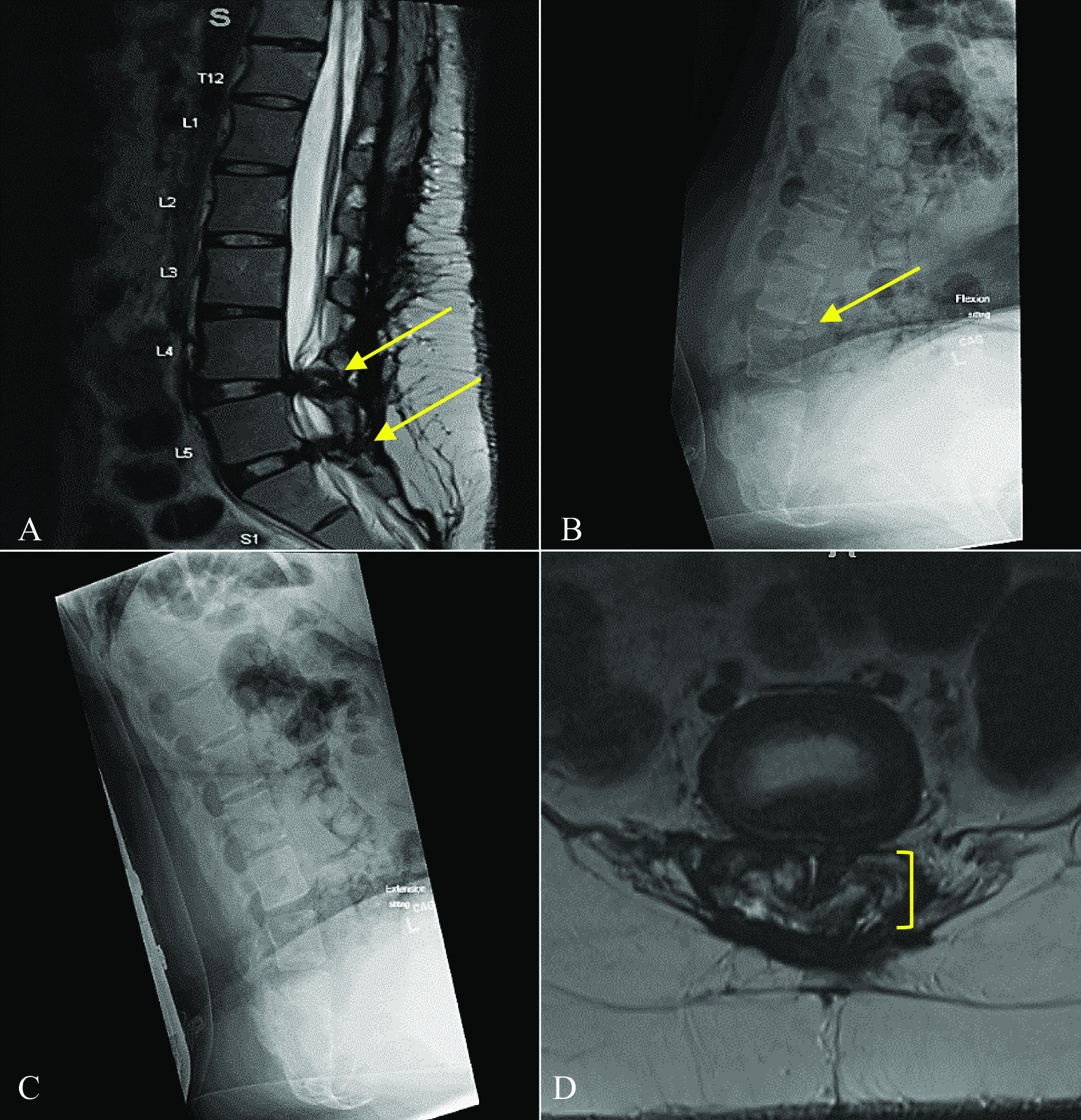
Preoperative images obtained prior to redo lumbar decompression and fusion surgery, ~ 80 months after diagnosis of TM and 30 months after removal of segmental instrumentation. **a** T2-weighted sagittal MRI of the lumbar spine demonstrating severe spinal stenosis at L4–L5 and L5–S1 (arrows) with near-total occlusion of the thecal sac. **b** Flexion lumbar radiograph showing loss of L4–L5 apposition without spondylolisthesis (arrow). **c** Extension lumbar radiograph showing improved alignment. **d** T2-weighted axial MRI of the L4–L5 disc space (bracket), demonstrating severe spinal stenosis

Magnetic resonance imagaing (MRI) of the lumbar spine demonstrated severe central spinal stenosis at L4–5 and L5–S1 due to facet and ligamentum flavum hypertrophy (Fig. 2a, d). Flexion–extension X-ray of the lumbar spine demonstrated axial distraction of the posterior edges of the vertebral bodies at L4–5 and L5–S1 on flexion (Fig. 2b) with alignment improving on extension (Fig. 2c).

Given her worsening pain with evidence of lumbar spinal stenosis and instability, she underwent redo L4–S1 decompression and fusion. She underwent L4 and L5 laminectomies with bilateral L4–L5 medial facetectomies and foraminotomies and posterior lateral lumbosacral fusion with bilateral pedicle screws at L4, L5, and S1. She tolerated the procedure well and endorsed improved pain compared with prior to surgery. She was discharged to home on postoperative day 3.

**Fig. 3:**
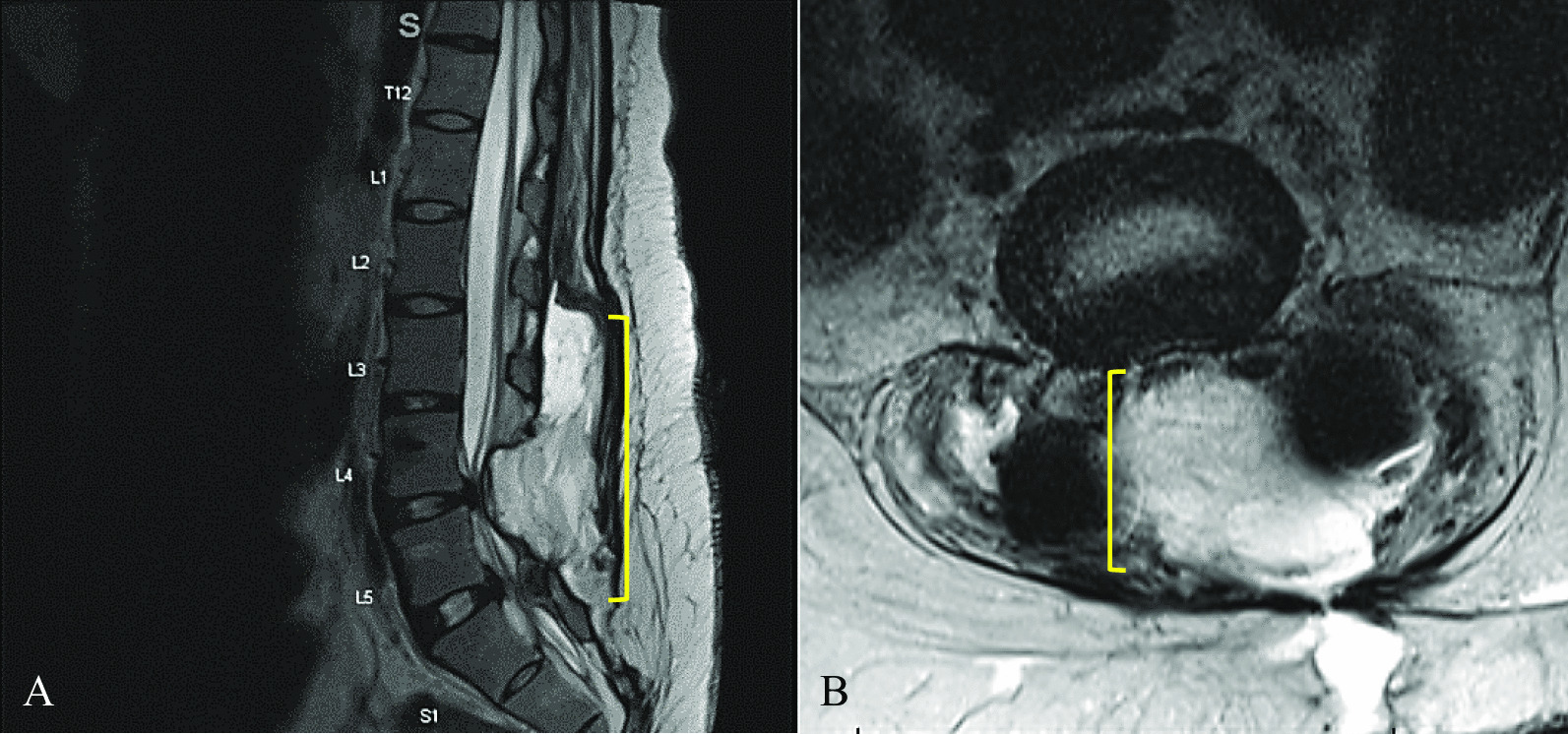
Postoperative images obtained 16 days after redo lumbar decompression and fusion surgery demonstrating surgical site infection. **a** Postoperative T2-weighted sagittal MRI showing surgical site fluid collection (bracket) compressing the thecal sac at L4–L5. **b** Postoperative T2-weighted axial MRI showing surgical site fluid collection (bracket) compressing the thecal sac at L4–L5

On postoperative day 16, she presented with fever, chills, tachycardia, and return of her midline low back pain. MRI demonstrated a surgical site infection with severe compression of the lower lumbar thecal sac (Fig. 3). Of note, the patient reported severe midline low back pain similar to what she experienced prior to surgery, identifying this as a separate pain from her postoperative soreness.

The patient underwent lumbar wound washout with significant improvement in low back pain. During her convalescence, she experienced incisional pain but reported significant improvement in her deeper, chronic pain, with an increased ability to perform transfers.

**Fig. 4:**
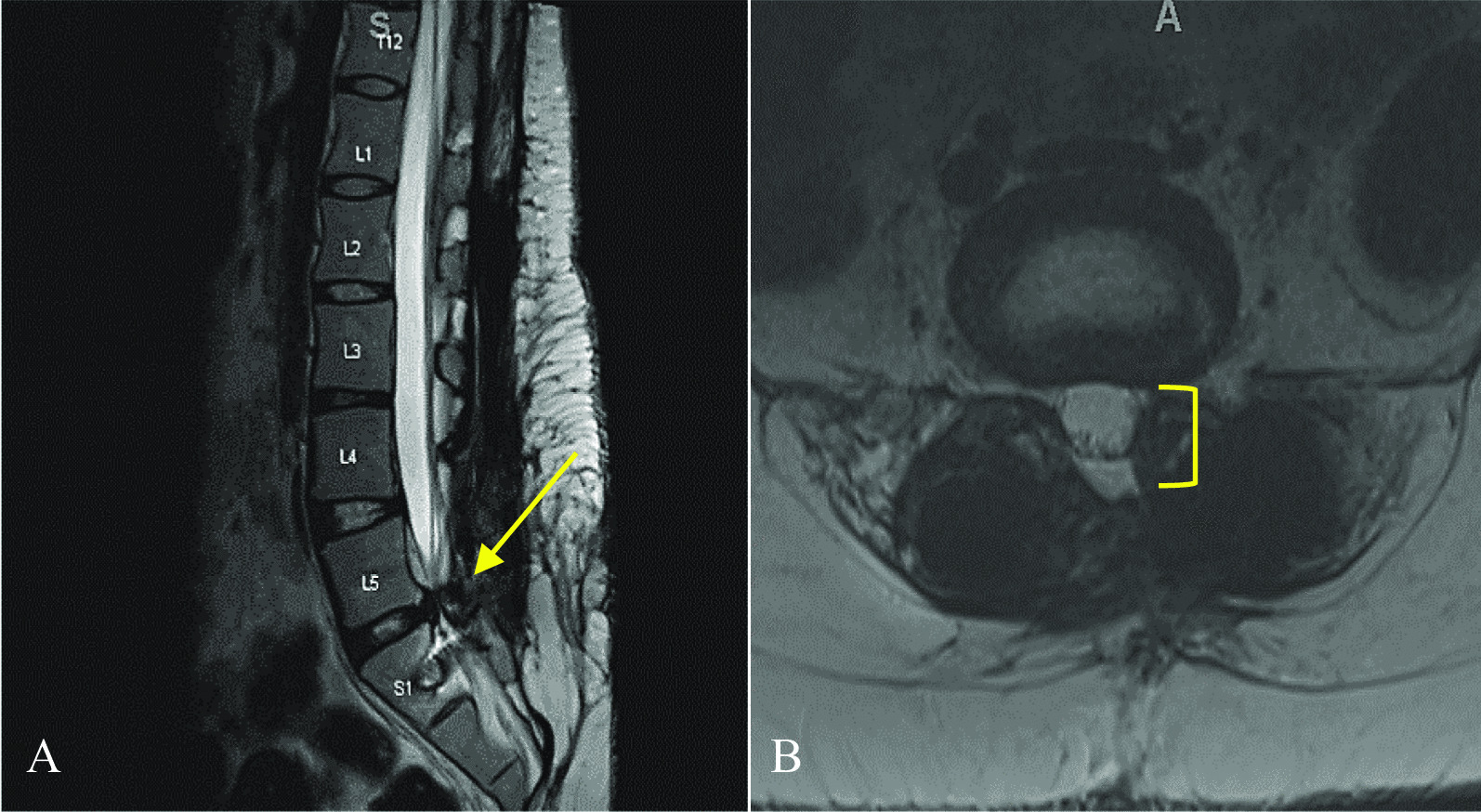
Follow-up images obtained 1 year following redo lumbar decompression and fusion surgery and subsequent washout. **a** T2-weighted sagittal MRI at 1-year postoperative, demonstrating decompression of L4–L5 level but with increased stenosis at L5–S1 (arrow). **b** T2-weighted axial MRI showing decreased stenosis (bracket) at the L4–L5 level

At 1 year postoperative, the patient’s chronic low back pain remained improved compared with preoperative baseline. The pain was described as “moderate” and “not as severe as preoperative back pain”. The patient reported doing well, with improved ability to bend and overall mobility. Follow-up MRI demonstrated decompression at L4–L5. Imaging also demonstrated increased spondylosis at L5–S1 (Fig. 4). Repeat decompression at L5–S1 with revised fusion at this level was offered, but the patient ultimately decided not to proceed with surgery as her back pain was no longer severe.

## Discussion

In the current case, our patient experienced chronic back pain below a thoracic sensory level after an episode of transverse myelitis (Fig. 1). While patients with TM are known to suffer from chronic neuropathic pain below a sensory level, our patient’s pain was typical of mechanical low back pain and responded, twice, to surgical decompression. Pain burden scores (Table 1) before and after surgeries demonstrate improvement in pain from 22 to 13, from 22 to 18, and from 18 to 12 in the first decompression surgery, pelvic fixation surgery, and removal of segmental instrumentation surgery, respectively. While a PBS score was not obtained following the second decompression and fusion surgery, the patient reported a subjective improvement in low back pain. These data support that decompression surgery improved mechanical low back pain below the level of TM.

**Table 1:** Surgeries since episode of transverse myelitis

Surgery	Time since initial episode of TM (months)	Pre/postoperative	Pain burden score (low back)
Posterior spinal fusion T3-pelvis	23	Preoperative	22
		Postoperative	13
Pelvic fixation	45	Preoperative	22
		Postoperative	18
Removal of segmental instrumentation	50	Preoperative	18
		Postoperative	12
Redo lumbar decompression and fusion	80	Preoperative	15
		Postoperative	Not obtained^a^

Pain burden score is scored 0–28, with 28 most severe low back pain

^a^PBS score was not obtained following redo lumbar decompression and fusion. However, patient did report subjective improvement in low back pain

Additionally, following redo decompression and fusion, postoperative thecal sac compression at L4–5 due to surgical site infection led to reemergence of pain similar in quality and intensity to her preoperative back pain, and wound washout with decompression of the thecal sac once again resulted in resolution of her pain. Mechanical instability in the lumbosacral spine may have also contributed to preoperative back pain, but the patient’s hardware remained in good position and, thus, likely was not involved in the postoperative pain that resolved with lumbar washout. This provides additional support to our hypothesis that our patient was symptomatic with somatic pain from lumbar spinal stenosis rather than post-TM neuropathic pain.

Overall, we believe that this patient’s chronic, somatic pain in the setting of a thoracic sensory level can be explained by severe, chronic compression of spinothalamic pathways that were preserved or regenerated following her episode of TM.

Several reports describe interventions for chronic pain after TM beyond typical analgesic pharmacotherapy and physiotherapy. These interventions include spinal cord stimulation [7–12], intrathecal morphine pump therapy [13, 14], acupuncture [15], and scrambler therapy (a technique involving transcutaneous nerve stimulation of the spinal cord) [16]. The patients described by these groups experienced chronic neuropathic pain with paresthesia, typically involving the legs, trunk, and arm(s). All of these patients experienced considerable disability and reduced quality of life due to their pain, and these metrics improved when their pain level was reduced via the above interventions.

In contrast to our patient, none of these patients had spinal cord compression on imaging. While many of the reported patients had paresthesia as a sequela of TM, none had complete sensory loss and none had focal low back pain, unlike our patient. Our patient did have marked improvement in her quality of life and functional status after surgery. We believe that following the patient’s episode of TM, aberrant signaling resulting from axonal regeneration and subsequent compression of these aberrantly organized tracts caused pain.

This case supports the clinical distinction and differences in management between chronic neuropathic pain due to TM versus chronic somatic pain due to spinal compression. As this is a case report, additional evidence is needed to make stronger conclusions regarding the causes of low back pain in TM, including, but not limited to, further basic science investigation that may explain the pathophysiology of pain signaling in transverse myelitis and larger cohort studies investigating the severity and quality of back pain before and after decompression surgeries following episodes of TM.

## Conclusion

To our knowledge, this is the first reported case of a patient with symptomatic lumbar stenosis after TM whose lower back pain and quality of life improved following surgical decompression and fusion. This case provides evidence that typical lumbago is possible in patients with sensory loss from TM, and standard lumbar decompression may provide benefit for these patients.

## Data Availability

Not applicable.

## Abbreviations

TM: Transverse myelitis
PBS: Pain burden score
